# Paired Trial Classification: A Novel Deep Learning Technique for MVPA

**DOI:** 10.3389/fnins.2020.00417

**Published:** 2020-04-30

**Authors:** Jacob M. Williams, Ashok Samal, Prahalada K. Rao, Matthew R. Johnson

**Affiliations:** ^1^Department of Computer Science and Engineering, University of Nebraska-Lincoln, Lincoln, NE, United States; ^2^Department of Mechanical Engineering, University of Nebraska-Lincoln, Lincoln, NE, United States; ^3^Department of Psychology, University of Nebraska-Lincoln, Lincoln, NE, United States

**Keywords:** EEG, MVPA, deep learning, machine learning, cognitive neuroscience

## Abstract

Many recent developments in machine learning have come from the field of “deep learning,” or the use of advanced neural network architectures and techniques. While these methods have produced state-of-the-art results and dominated research focus in many fields, such as image classification and natural language processing, they have not gained as much ground over standard multivariate pattern analysis (MVPA) techniques in the classification of electroencephalography (EEG) or other human neuroscience datasets. The high dimensionality and large amounts of noise present in EEG data, coupled with the relatively low number of examples (trials) that can be reasonably obtained from a sample of human subjects, lead to difficulty training deep learning models. Even when a model successfully converges in training, significant overfitting can occur despite the presence of regularization techniques. To help alleviate these problems, we present a new method of “paired trial classification” that involves classifying pairs of EEG recordings as coming from the same class or different classes. This allows us to drastically increase the number of training examples, in a manner akin to but distinct from traditional data augmentation approaches, through the combinatorics of pairing trials. Moreover, paired trial classification still allows us to determine the true class of a novel example (trial) via a “dictionary” approach: compare the novel example to a group of known examples from each class, and determine the final class via summing the same/different decision values within each class. Since individual trials are noisy, this approach can be further improved by comparing a novel individual example with a “dictionary” in which each entry is an average of several examples (trials). Even further improvements can be realized in situations where multiple samples from a single unknown class can be averaged, thus permitting averaged signals to be compared with averaged signals.

## 1. Introduction

Deep learning has produced state-of-the-art results in many areas of machine learning, but adoption of deep learning for the classification of electroencephalography (EEG) signals, and other types of human neuroscience datasets, has lagged compared to its popularity in other fields. Although an increasing number of studies are using deep learning to process neuroimaging datasets, the improvements in performance have typically not been as drastic as in other fields (Lotte et al., 2018), and most human neuroscience research has continued to use more traditional multivariate pattern analysis (MVPA) approaches: Manual feature extraction followed by a simple, typically linear, classifier, such as support vector machines (SVMs; Cortes and Vapnik, 1995) or logistic regression and its derivatives, e.g., sparse multinomial logistic regression (SMLR; Krishnapuram et al., 2005).

Nevertheless, deep learning techniques are being explored in EEG classification. Bashivan et al. (2015) used a recurrent convolutional model to classify EEG data that was projected onto a two-dimensional plane and then subjected to Fourier analysis. The final model achieved an error rate of 8.89%, as compared to a 12.59% error rate with a random forest. While this is a meaningful reduction in error rate, boosting was not employed in the training of the random forest, which likely would have significantly shrunk the difference in performance. Lawhern et al. (2018) explored the use of fully convolutional neural networks; they applied convolutions in data that were arranged in a (channels × timepoint) fashion to create a two-dimensional matrix. These models had very few features, on the order of 2,200. This work showed improvements over the Filter Bank Common Spatial Pattern algorithm in a majority of the datasets tested, including the P300 event-related potential (ERP) in an oddball task, error-related negativity in brain-computer interfaces, movement-related cortical potential in a finger movement task, and sensory motor rhythm in imagined movement. Schirrmeister et al. (2017) further demonstrated the applicability of convolutional neural networks in decoding raw EEG signals without hand-crafted features. They showed that the learned filters were able to extract information in the alpha, beta, and high gamma wavelengths, and found a small improvement over the Filter Bank Common Spatial Pattern algorithm in their test dataset (82.1% accuracy to 84.0%) accuracy.

There are many possible reasons for modern deep learning techniques to underperform in EEG classification, compared to the drastic benefits deep learning has had for other fields. For one thing, EEG data are very noisy. The electrical activity that makes it to the recording electrodes is spatially smoothed and otherwise distorted by passing through poor conductors, such as the skull and scalp. Signals propagating in opposite directions interfere with each other and reduce the signal that makes it to the sensor. Even more importantly, human subjects' cognition and brain activity naturally fluctuate from trial to trial; on some trials, they may not be focused on the task at all, and thus may produce brain signals that poorly reflect the trial type they are presented with. As such, if participants' attentiveness cannot be inferred from behavioral performance, some trials may not be classifiable at all, despite lacking any overt signal artifacts. While averaging can be used in some cases to reduce the impact of this unclassifiable data, it is not practical in all situations, such as when working with brain-computer interfaces where real-time, single-shot classification is the ultimate goal.

EEG data also have very high dimensionality. Signals in most cognitive neuroscience studies are generally recorded with a sampling rate of between about 250 and 1,000 Hz, with anywhere between about 16 and 256 channels of data. Overfitting is common on such high-dimensional signals. This problem is further exacerbated by the limited number of examples (trials)^1^ that are usually available. It is impractical to collect EEG datasets on the scale of hundreds of thousands of examples, as seen in other deep learning applications, such as image classification, as this would require extraordinarily long recording sessions with human subjects and/or an unreasonably large number of them. Finally, this is all further compounded by the large individual differences between different human subjects (e.g., Valenzi et al., 2014). While a digital image of, say, a traffic light could be taken from many different angles, under many different lighting conditions, etc., traffic lights still have a number of visual properties that are presumed to be more-or-less invariant across different conditions and exemplars; if a so-called “traffic light” were shaped like a pyramid, gelatinous, and translucent, and contained lights of blue/magenta/orange, most image classifiers (including human beings) would fail to recognize it as such, but it could also rightly be argued that those changes make it no longer a true “traffic light” anyway. In comparison, it is much more difficult to make such distinctions in patterns of neuroscience data across human beings; while certain general phenomena appear to be near-universal across most humans, such as the N170 ERP to face stimuli (Bentin et al., 1996), there is still substantial variation across individuals and trials that can be sufficient to fool many classifiers. And, because it is usually impractical to determine whether these variations are due to differences in head shape, recording artifacts, fluctuating attention, functional brain organization/connectivity, cognitive strategy, etc., it is much more difficult to establish any kind of ground truth as to what an ideal response would look like. Suppose a human participant exhibits no N170 ERP but has intact face recognition ability, with no discernible artifacts in their data; how do we reconcile this? In the EEG data, we have the equivalent of a pyramidal, gelatinous “traffic light” but are confronted with the awkward task of trying to determine if we can possibly align it, somehow, with all the other pictures of rectangular solid ones.

Even if deep learning has not yet produced drastic improvements in classification performance relative to traditional MVPA techniques for most cognitive neuroscience applications, it is still worth exploring further; there are a tremendous number of possible configurations of deep neural network architectures, and thus far we have only scratched the surface of what might be possible with them. However, if we do want to increase performance in the analysis of neuroscience data with deep learning, it might be wise to begin thinking about ways of changing how we could reformulate the basic problem. This paper describes one such possible reformulation (out of probably many): Instead of classifying a single example at a time, one could instead attempt to classify *pairs* of examples as belonging to either the same class or different classes. We refer to this general approach as paired trial classification (PTC), described further in section 2.2.2. This method presents several potential benefits. First, it allows for a drastic increase in the number of training examples, as there are *O*(*n*^2^) possible pairs. This makes it easier to find a neural network model that reliably converges, which can be a significant issue in datasets with a comparatively large number of features but comparatively few examples. Also, given the otherwise low impact of standard data augmentation techniques in the field (Bashivan et al., 2015), PTC could also potentially improve the ability of the model to generalize to new data by reducing the likelihood of the model to memorize samples from the dataset. Second, it reduces the problem to two classes, potentially simplifying multi-class problems and thus presenting a second way of making it easier to achieve robust classification performance from limited training data. Third, it is flexible: The basic same/different judgment can be interpreted either categorically or continuously, as a kind of similarity metric; it can be combined with a “dictionary” approach (see below) to achieve traditional multiclass classification; and trained PTC models can in principle be used with any input data, not necessarily just the categories it was initially trained with, which could have interesting theoretical applications in the future.

As alluded to above, when trying to classify a novel example into one of several categories, PTC could still be used by employing a “dictionary” approach. That is to say, a new trial can be compared to known trials from all known classes and classified as the same class as the exemplar(s) to which it is/are most similar. Thus, for a single trained network model, this allows us to classify each example in the test set multiple times and average the results of those individual decisions into a single overall classification, which has the potential to reduce variability in classification performance for individual trials. Novel trials could also be compared against averaged signals from multiple trials drawn from the training set. This allows for more stability in the comparisons, further addressing the issue of noise in EEG signals. Similar approaches based on “dictionary” comparisons and/or averaging have been used with traditional MVPA going back to its neuroimaging roots (Haxby et al., 2001), but PTC allows those approaches to be combined with the power and flexibility of deep learning.

Ideas similar to PTC, also with an intent to increase the size of the dataset and the accuracy of the classifier, have been explored in other domains. A similar pairing technique has also been explored, but at the pixel-classification level, in hyperspectral imaging. Rather than classifying individual pixels, Li et al. (2016) classified a pixel in combination with each of its neighboring pixels and used a voting strategy to determine the class of the original pixel. Another similar approach by Inoue explored data augmentation through the unweighted averaging of two images in the training set. These images were not required to be drawn from the same class but were always given the label of the first chosen image, thus preventing perfect memorization of the data. The final fine tuning was performed on unaugmented data. They demonstrated substantial performance improvements on the ILSVRC 2012 and CIFAR-10 datasets using GoogLeNet. This approach differs from our proposed approach in that it performs averaging rather than concatenation, and does not attempt to predict the sameness of the two samples (Inoue, 2018). Using a technique termed “Matched Pair-Learning,” Theiler (2013) paired two signals with statistical dependence but differing labels (e.g., different frames of chemical plume data) and classified the pair together, but their aim was not to classify whether those signals had the same or different category, *per se*. Comparisons of trials of neuroscience data to each other, or to averaged sets of trials, have also been relatively commonly applied in “pattern similarity” analyses within the traditional MVPA domain of cognitive neuroscience; in essence, our method is an enhanced version of those pattern similarity approaches, which would typically use Pearson correlation, Euclidean distance, or other distance/similarity metrics (for more, see section 2.2.2 below). However, to our knowledge, this is the first time such an approach has been tried within the domain of deep learning, or conversely, the first occasion in which deep learning has been applied in the domain of pattern-similarity MVPA to achieve a similarity metric customized to the dataset at hand, and thus one that is “smarter” than existing metrics based purely on mathematical formulas.

## 2. Materials and Methods

### 2.1. Data

**Figure 1:**
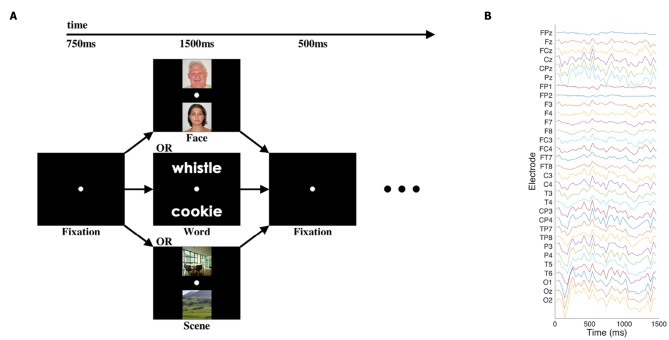
Cognitive task and sample EEG data. **(A)** Participants viewed pairs of one of three categories of images at the beginning of each trial of the cognitive task, with blank-screen fixation intervals before and after. Other task components followed the presentation of the images, but those elements of the task are not presented or analyzed here. **(B)** Single representative trial of EEG data after pre-processing and downsampling. Electrode labels are according to the standard 10–20 and 10–10 systems for EEG electrode placement.

We used an EEG dataset consisting of the “initial presentation” period of a cognitive neuroscience study first published by Johnson et al. (2015); for full details, please see that paper. Briefly, in the pertinent portion of that study, participants were presented with a pair of visual stimuli for 1,500 ms: either two written words, two images of faces, or two images of scenes. Thirty-one channels of EEG data were recorded at 250 Hz. The signals were bandpass-filtered via hardware in a 0.01–100 Hz range, and recorded with 14 bits of precision. See Figure 1 for an illustration of the stimuli and data.

A total of 37 subjects participated in the study and had high enough quality data to be used. We used the same initial pre-processing steps, trial rejection parameters, and participant exclusion criteria as described originally by Johnson et al. In the original publication, there were two experiments with *N* = 21 and *N* = 16, but the “initial presentation” period did not differ between experiments, and thus we have combined them into a single *N* = 37 dataset for present purposes. Each subject had ~200 trials after artifact rejection (around 60–70 per image category), for a total of just under 7,000 trials.

The data were then subjected to additional pre-processing for the deep-learning-based PTC analyses. All data values (originally in raw microvolts) were divided by a fixed factor of 20 to bring their scale approximately into the −1 to 1 range in which neural networks perform the best. Additionally, time averaging was applied to reduce the dimensionality of the data by a factor of 10, i.e., data were downsampled into time bins of 40ms apiece, similar to the bins used for MVPA in the original publication (Johnson et al., 2015). Thus, the total data dimensionality per trial was 31 *channels* × 37 *time bins* = 1, 147 *features*.

### 2.2. Classification Methodology

#### 2.2.1. Baseline Models

In order to attain a baseline classification accuracy on the dataset, several widely used classifiers were examined. These include both a traditional classification baseline and a deep learning classification baseline. These models were trained on both single trials and averaged trials to allow for comparisons between the PTC methodology and other established techniques.

Traditional classification baselines were set using SVM and SMLR techniques, which are both frequently applied in traditional MVPA^2^. SVM analyses used a linear kernel, which is also common in neuroscience studies using MVPA, and which we have found to outperform radial basis function kernels in some of our previous analyses of EEG data. SVM hyperparameters were chosen with grid search over C in the set [0.0001, 0.001, 0.01, 0.1, 1, 10, 100]. Similarly, the lambda hyperparameter in SMLR was chosen through grid search from the set [0.001, 0.01, 0.1, 1, 10, 100, 1000]. These value ranges were chosen to span a range commonly seen in practice, in order to ensure that our comparisons were as fair as possible to the baseline conditions (i.e., that we did not hamstring the baselines by a poor choice of hyperparameter).

The deep learning model developed for paired-trial classification (see below) was also used for baseline analyses as a more conventional three-class neural network classifier (deep neural network [DNN] baseline model). This was done by slightly altering the network architecture, namely, by modifying the input layer to accept a single trial (rather than a pair), and modifying the output layer to have three output nodes instead of two, while leaving all hidden layers the same between the baseline DNN and PTC network architectures. It is certainly possible, given the effectively infinite number of combinations of architectures and hyperparameters, that better-performing DNN models could be found for the baseline analysis and/or the PTC analysis; however, for this initial demonstration of PTC we simply chose one relatively straightforward model that we thought would be fairly representative of the types of DNNs used to analyze cognitive neuroscience data.

#### 2.2.2. Paired Trial Classification (PTC) Technique

The essence of the PTC approach is that instead of training a neural network to classify a single trial of data into one of several classes, the network is instead trained to determine whether two trials of input data are drawn from the same class or different classes. This binarization of the problem is somewhat different to the approach of, say, performing multiclass classification with SVMs by creating a number of binary SVMs and summing their outputs, because with the PTC approach, the same network can theoretically learn to classify similarities or differences between pairs of trials drawn from any class, for theoretically any number of classes. As such, PTC essentially gives us a new kind of similarity/distance metric with some useful characteristics: It can be interpreted either as a categorical same/different judgment or a continuous similarity/dissimilarity score, and it is “smarter” than simple formula-based metrics, such as Euclidean distance, cosine similarity, and Pearson correlation, having been trained to be sensitive to the features of a specific dataset that matter in differentiating the classes, while ignoring any nuisance features. To do this, each example fed into the classifier is comprised of two trials of EEG data (with dimensions Samples × Channels), stacked together to form a 2 × Samples × Channels input. Regardless of how many classes or conditions are present in the original dataset, the output layer always has two units, one representing a decision that the two trials in the input example are drawn from the same category, and the other unit representing a decision that the two trials are drawn from different categories. This also means that, in theory, a trained PTC network could be applied or adapted (via transfer learning) to previously unseen categories, although in this initial demonstration we do not yet test that possibility.

We explored three variations of PTC analyses:

1. *Single-to-single*: In our initial analyses, we perform PTC using pairs of individual (un-averaged) trials. This variation will be referred to as “single-to-single.” One difficulty in performing single-to-single PTC is that when individual trials are relatively noisy or variable, as is often the case in neuroimaging data and EEG in particular, the problem is compounded by directly comparing two single recordings. Thus, performance, in this case, might be expected to be worse than typical neural network classification. By way of comparison, although traditional DNNs are trained and tested on individual trials, the network itself effectively embodies the features that worked best across the full breadth of the training set. In that sense, traditional DNNs might be a closer comparison in some ways to the single-to-average PTC analysis (see #3 below). To address the noise issue that arises when comparing two individual trials, we also performed PTC using two approaches that incorporate some form of averaging prior to classification, and use trial averages rather than individual trials as one or both elements of each input to the model.
2. *Average-to-average*: In the first averaging-based PTC variation, each training/validation/test example was composed of two trial averages that combined signals from 20 trials each, without overlap. This variation will be referred to as “average-to-average.” At this point, it is important to note that most analyses are performed under the assumption that we have a pre-existing “known” dataset and a novel “unknown” dataset. As such, we explore the case in which the first signal in the pair is composed of an average generated from a single “unknown” subject, and the second signal is generated from an average across multiple “known” subjects, to form a sort of exemplar pattern. For further details, see section 2.4.
3. *Single-to-average*: In this analysis, a single trial was compared to a 20-trial average. This variation will be referred to as “single-to-average.” Again, this is performed with the assumption of “known” and “unknown” datasets. The single trial is drawn from the “unknown” set, while the averaged trial is computed across multiple subjects from the “known” dataset. Again, see below for further details.

#### 2.2.3. Dictionary Approach

The basic same/different PTC classifier can readily be mapped to multiclass classification with the use of a corpus, or *dictionary*, of “known” trials. To classify a given “unknown” sample, it is compared to sets of “known” trials from each class using PTC. The likelihood scores for each of the classes are passed through a softmax function (Bridle, 1990), and the classification decision for the unknown sample is determined by whichever dictionary class has the highest average softmaxed likelihood value.

A naïve implementation of dictionary selection was used. One hundred trials from each of the three classes were chosen at random from the test and validation set to form the dictionary. In the approaches that compare against averaged signals, each of the trials in the dictionary is created by averaging 20 randomly selected signals from the same class, chosen across subjects.

### 2.3. Network Architecture

**Figure 2:**
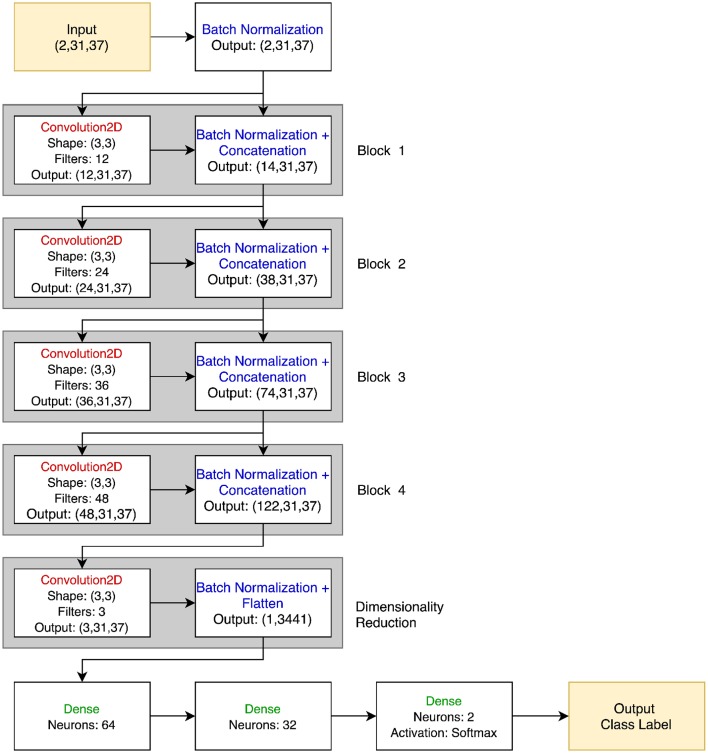
PTC neural network architecture. The input is two 31 × 37 EEG signals, stacked together in a 2 × 31 × 37 3D array. Batch normalization is immediately applied to each signal, and the output of this is both passed through the 12 filter banks in the Convolution2D layer of Block 1 and passed directly to the Batch Normalization block. Thus, the Batch Normalization layer of Block 1 outputs 12 + 2 = 14 images of size 31 × 37. This process is repeated for a total of four blocks. No concatenation is performed in the dimensionality reduction block, and the 3 × 31 × 37 feature map is flattened and passed to the final dense layers before classification. All convolutional and dense layers use the Leaky ReLU activation function unless otherwise specified.

Although in principle any number of architectures could be used for PTC, a straightforward choice for this initial demonstration was to use a convolutional network model, as that afforded a clear way of training a classifier that could learn to compare its two inputs. Each of the two trials is treated as an input channel, so convolution can naturally capture their parallel (both channel and time) nature^3^. A 3 × 3 2D filter was chosen to act over both channels and time, respectively, using zero-padding to maintain signal dimensionality between layers. The results were passed through a leaky rectified linear unit activation (Leaky ReLU) function and then a batch normalization layer. Four blocks of convolution, activation, and batch normalization layers were used. The number of filters per block was increased successively from 12 to 48 to capture the hierarchical nature of the feature representations. Each block was connected not only to the next block with the output of the batch normalization layer feeding into the next convolution, but all subsequent blocks with skip connections using concatenation, as in DenseNet (Huang et al., 2017). At the end of the densely connected portion, a final 2D convolution with three filters was applied to reduce the data dimensionality before feeding it into two fully-connected layers with 64 and 32 neurons, and then a final classification layer. In total, the network had 246,909 trainable parameters. While the total number of parameters is substantially smaller than found in many deep networks, previous literature has suggested that substantially down-scaled neural networks are appropriate for neuroimaging data, in part due to the tendency of larger networks to overfit when trained with the limited size of dataset available in neuroimaging (Bashivan et al., 2015). See Figure 2 for a visual representation of the PTC network.

As noted above, a similar network was used for the baseline DNN model that used a more conventional three-category classification approach. The only differences were that in the baseline DNN's network, the first convolutional layer only accepted a single trial's data, and the final layer had one output per class. This version of the network thus had a very similar 245,862 trainable parameters (less than half a percent fewer than the PTC network), as the vast majority of the parameters are found in the dense layers, which share the same input and output shapes between the two models.

### 2.4. Training, Validation, and Test Procedures

In all the variations of PTC, subjects were split into three disjoint groups: training, validation, and test. A leave-one-subject-out cross-validation methodology was used, and the remaining subjects were split with 80% randomly assigned to training, and the remaining 20% assigned to validation. As per standard, the training group was used to perform backpropagation updates on the models; the validation group was used to determine a stopping criterion for updating the model, and the test group was used to determine the accuracy of the models.

All models were trained using the Adam optimization algorithm (Kingma and Ba, 2015) and a batch size of 144. Minibatches were generated dynamically during training. Samples were drawn randomly from all subjects in the training group, with an even split across all possible class pairs and orderings (e.g., Face-Face or Scene-Word). Since, in a three-class problem, there are six “different” pairings and three “same” pairings, the “same” pairings were sampled twice as often to provide equivalent numbers of “same” and “different” pairings during training. In the average-to-average analysis, averages were constructed such that no trials were shared between the two averages; in single-to-average, the single trial was never one of the trials used to comprise the average.

Standard techniques were implemented to reduce the likelihood of overfitting. Dropout was enforced on the dense layers (Srivastava et al., 2014), with a proportion of 10%. We initially tried architectures with higher dropout rates, which would be more standard usage of the dropout algorithm, but those rates resulted in reduced performance during training across all analyses and unreliable training convergence in the single-to-single PTC variation. Early stopping was employed when validation loss failed to improve for a period of 30 epochs. The model from the epoch in which the lowest validation loss was observed was chosen as the final model.

For single-to-single and single-to-average “same-different” accuracy, each sample in the test set (i.e., held-out subject) was compared to a randomly selected set of trials drawn from the training and validation sets, with 100 signals per class, as described in section 2.2.3. For the average-to-average PTC method, 80 averages were generated per class from the test set (roughly approximating the number of individual trials per class that a single subject would have) and then compared against a dictionary built from the training/validation sets as in the other PTC analyses.

For the baseline (non-PTC) deep learning analyses, a similar leave-one-subject-out cross-validation scheme was used, with the same network hyperparameters as the PTC analyses (optimization algorithm, dropout, early stopping, etc.). Similar to PTC, the non-left-out subjects were split with 80% randomly assigned to training, and the remaining 20% assigned to validation. For all deep-learning-based analyses, ten iterations of the cross-validation were performed per left-out subject, and results from all iterations were averaged to yield the final results we present below.

SVM and SMLR models were also tested using leave-one-subject-out cross-validation, but without a validation set. That is, the models were trained on trials from all but one subject, and the remaining subject's trials were then used for testing.

### 2.5. Environment

All deep learning analyses (PTC analyses + the DNN baseline analysis) were performed in Python 3.6 using the Keras toolbox (Chollet, 2015) with a Theano backend (Theano Development Team, 2016). Custom in-house Python scripts were used to implement the specific analysis techniques we used, tabulate results, and so on. NumPy was used in supporting functions (Oliphant, 2006; Walt et al., 2011). SciKitLearn was used for the SVM (Buitinck et al., 2013) and PyMVPA was used for SMLR (Haxby et al., 2011).

## 3. Results

### 3.1. Base Models

The performance of the three baseline classifiers is shown in Table 1. All values reported are derived by first calculating mean accuracies for each human participant (averaged across iterations of the cross-validation algorithm, for all deep learning models; SVM and SMLR are deterministic and did not require multiple iterations), and then averaging across participants. Overall, SMLR (with a lambda parameter of 100) achieved the highest performance on single trials, with an accuracy of 64.90% (against chance = 33.33%). SVM (with a C parameter of 0.0001) achieved the second-best results with an accuracy of 63.67%. Finally, the DNN model achieved an accuracy of 59.51%. SMLR's performance was significantly better than SVM's (*p* = 0.0064) and SVM's was significantly better than the DNN model's (*p* < 10^−7^; all comparisons are paired *t*-tests).

**Table 1:** Baseline accuracy (percent, with chance = 33.33%).

	**SVM**	**SMLR**	**DNN**
Single	63.67	**64.90**	59.51
Averaged	81.54	82.52	**82.54**

*Bold values indicate highest accuracy in each row*.

In the averaged-trials condition, all baseline models performed similarly. The DNN model performed infinitesimally better than SMLR, at 82.54 and 82.52%, respectively. SVM achieved a slightly lower accuracy of 81.54%. However, none of these values were significantly different from each other (all *p* > 0.3).

### 3.2. PTC

The overall PTC results are shown in Table 2. The same-different classification had a chance accuracy of 50%, and the dictionary classification approach had a chance accuracy of 33.33%. Generally, as more averaging was applied, the accuracy increased. Same-different accuracy improved from 56.03% (single-to-single) to 71.25% (single-to-average) to 86.15% (average-to-average) as the averaging was increased. As expected, all of these values were significantly different from each other (all *p* < 10^−18^).

**Table 2:** PTC accuracy summary (percent).

	**Same/Different**	**Dictionary**
Single-to-Single	56.03	49.21
Single-to-Average	71.25	61.53
Average-to-Average	86.15	83.32
(Chance)	50.00	33.33

Similarly, dictionary classification improved with more averaging, from 49.21 to 61.53 to 83.32%. Again, as expected, all of these values were significantly different from each other (all *p* < 10^−16^).

The single-to-single PTC dictionary classification performed worse than all of the single baselines (*p* < 10^−13^ against all single-trial baseline classifiers). Similarly, the single-to-average PTC dictionary model performed worse than all of the averaged-trial baselines (all *p* < 10^−11^). However, given the differences in the algorithms, a “fairer” comparison might be between the single-to-average PTC dictionary model and the single-trial baseline classifiers, since the trained baseline classifiers implicitly contain a form of averaged representation of the training data, against which individual trials are compared during testing. The single-to-average PTC dictionary still performed significantly worse than the single-trial SVM and SMLR classifiers (both *p* ≤ 0.001), but it did outperform the single-trial DNN classifier with a nearly identical network architecture (61.53 vs. 59.51%; *p* < 10^−7^).

The average-to-average PTC dictionary classification did perform with a numerically higher accuracy than all of the averaged-trial baselines (83.32% for PTC vs. next-highest DNN baseline at 82.54%). However, as with the comparisons among the individual averaged-trial baseline models, the average-to-average PTC dictionary classifier was not significantly different from any of them (all *p* > 0.14).

The same-different confusion matrices for the three methods are shown in Table 3. All three models are more likely to predict that two samples came from a different underlying class than the same underlying class, with the difference being more pronounced as more averaging is involved. As a result, accuracy was higher when the actual trial pair was a “different” pair than when it was a “same” pair.

**Table 3:** Confusion matrices for PTC analyses (percent).

		**Single-to-Single**		**Single-to-Average**		**Average-to-Average**	
	**Predicted:**	Same	Different	Same	Different	Same	Different
**Actual**							
Same		53.71	46.29	60.96	39.04	79.39	20.61
Different		42.74	57.26	23.50	76.50	10.47	89.53

**Figure 3:**
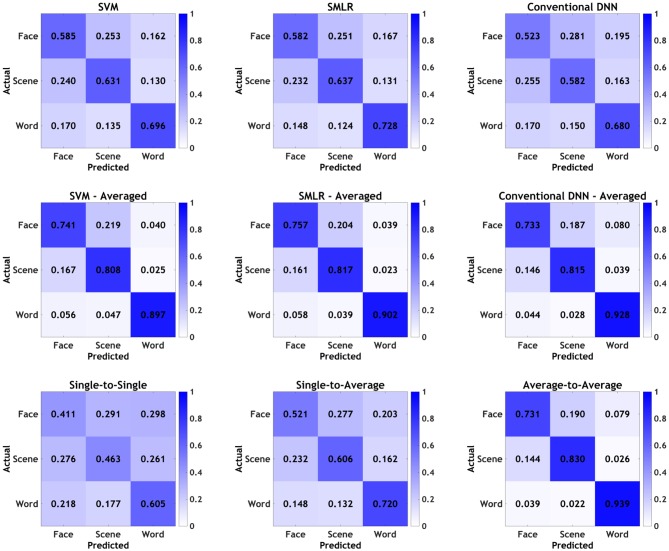
Confusion matrices by individual stimulus categories for all classifiers. Values presented as proportions rather than percentages for readability.

Finally, graphical confusion matrices of the individual stimulus categories are shown for baseline classifiers and PTC analyses in Figure 3. Broadly speaking, all classifiers showed the same general pattern, with words being correctly identified most often, followed by scenes, followed by faces. In all cases, averaging improved performance, and various individual classifiers performed better than others, as detailed above; however, none of the classifiers or manipulations appeared to show a qualitative difference in the pattern observed in the confusion matrices, beyond those that tracked with overall increases/decreases in accuracy. As such, it appears that, broadly speaking, all classifiers were picking up on approximately the same general patterns in the data, with no classifiers or manipulations appearing to show a particular bias for one category over the others.

## 4. Discussion

In this paper, we demonstrated a new method of deep-learning-based classification for neuroscience data, paired trial classification (PTC). Rather than using a DNN to classify a trial's category directly, we instead trained the classifier to compare pairs of trials to each other. Using a “dictionary” approach similar to ones employed in traditional MVPA studies with conventional distance/similarity metrics, we also used PTC to generate category predictions based on how often a test trial was judged to be the “same” as other trials drawn from the three categories of stimuli in our dataset. While it is difficult to draw direct performance comparisons between PTC and our baseline measures, given the significant differences in how the problem was structured and how the results could be interpreted, overall PTC performed comparably to other measures, and in some cases perhaps a bit better. Either way, we believe the novelty and flexibility of PTC make it an interesting approach and a viable avenue for future explorations into its potential.

In all cases, PTC performed with accuracy significantly above chance. While the same-different classification for the single-to-single paradigm was only marginally better than chance at 56.08% accuracy, the single-to-average paradigm was substantially better than chance with an accuracy of 71.32%. This represents the ability to say with some confidence whether a novel trial is similar to some exemplar formed from the averaging of known trials. Furthermore, the average-to-average paradigm is more accurate at 86.15% accuracy, allowing for a more confident determination of a group of novel samples known to be drawn from the same unknown class.

The tendency of the models to predict “different” more often than “same” is somewhat notable, considering the equal number of “same” and “different” examples provided during training. However, this tendency is straightforward enough to explain; it stands to reason that noise is more likely to make a trial appear as if it were coming from some different class than for noise to cause two trials from different classes to appear to be drawn from the same class. For instance, assume a research participant stops paying attention for one trial, or flutters their eyes enough to create a small artifact (but not one big enough to trigger rejection of the trial using standard preprocessing techniques). If the PTC algorithm is doing its job well, it is likely to judge that noise trial as being “different” from the trial it is paired with, regardless of whether the other trial is the same category or not. In that case, the PTC algorithm may not even be making an error when it judges some “same” pairs as “different”; instead, it might be picking up on unanticipated differences/noise in the data that are not accounted for by the comparatively simple assumption that all “face” trials, for example, should have similar neural activity to each other. This feature might be exploitable in future work; for example, to address the well-known issue with many conventional DNN analyses that deep networks often yield high confidence scores to noisy or adversarial inputs (Nguyen et al., 2015; Su et al., 2019), as implicit in their training is the tendency to maximize confidence scores as much as possible. In contrast, a PTC classifier given a poor input might correctly give high-confidence “different” responses to all of the possible categories (including the one that is nominally the same as the poor trial), which effectively can be read as a vote of no confidence in the quality of the input data.

We also observe that the dictionary-based classification technique allows for the successful mapping back to the multiclass classification paradigm. The results for the single-to-average dictionary classification condition were on par with any of the single trial baselines, and the average-to-average dictionary classification conditions were on par with the averaged signal baselines. These results were achieved with a naïve approach to dictionary selection, so better performance could be seen with the optimization of the dictionary; exploring potential improvements to the dictionary portion of the algorithm would be one promising direction for future work. Notably, the single-to-single paradigm stands to improve from a less noisy dictionary. As it stands in this implementation, a sort of “weak learner” effect is observed between the two applications of the single-to-single network: A single “same-different” classification was successful 56.08% of the time, only 6.08% above chance, but the three-class classification was successful 49.35% of the time, a more impressive 16.02% better than chance. Although the two values are not directly comparable given the differences in what they represent, they are suggestive that pooling the individually weaker “same-different” classifications across a multiclass dictionary can indeed produce robust overall results. This also raises the possibility that the PTC approach might be especially well-suited to datasets with higher numbers of classes. In particular, if some of those classes had too few trials in them to reliably train a conventional DNN to recognize them, a PTC network trained with trial pairs from all classes might still have a chance of picking them out.

Deep learning is useful because it can take advantage of the multi-dimensional nature of datasets in a way that other methods cannot (as the simpler linear techniques require vectorized input); GPU acceleration and parallelization in general are better supported for deep learning, making it more computationally efficient for large datasets; and deep networks can be configured flexibly to address a wider range of problem domains than simple linear methods. However, deep learning is frequently difficult to apply successfully in neuroscience. Often, human neuroscience datasets can fall into a “Goldilocks problem” zone, meaning that they can have too many trials or features for SVMs or other conventional MVPA approaches to be performant, but fewer training examples than are typically expected to enable DNN-based analyses to converge reliably. In such cases, data augmentation techniques could be applied to enable the use of deep learning by increasing the generalizability of the network. However, direct augmentation techniques pose challenges of their own. For example, Bashivan et al. (2015) found that temporal shifting techniques that have been applied successfully in other fields did not meaningfully improve generalization in their deep learning analyses of an EEG dataset. PTC offers an alternative way of increasing the size of the training dataset, not by augmenting the trial data itself, but rather by pairing trials combinatorially. However, it does not require altering the underlying data (except, optionally, by averaging trials to increase signal-to-noise, as we did here), which could be a useful property of this technique in specific scenarios, or provide another option to try when standard data augmentation techniques fail.

The goal of this paper was to introduce the PTC paradigm and show that it can easily be mapped back to multiclass classification. This approach is not limited, however, to cases in which there are a discrete, known set of classes as in typical classification applications. PTC could also be used for detecting trials that differ substantially from those seen in the training dataset, such as in outlier detection, novel stimulus identification, or artifact rejection. It could also be used in situations wherein a more conventional distance/similarity metric might be applied; for example, to assess neural pattern similarity across exposures to a set of stimuli, and to use these similarity judgments to test hypotheses about memory, make predictions about which stimulus is being seen or imagined at a particular point in time, or perform clustering analyses.

## Data Availability Statement

The datasets generated for this study are available on request to the corresponding author.

## Ethics Statement

The studies involving human participants were reviewed and approved by Yale University Institutional Review Board. The patients/participants provided their written informed consent to participate in this study.

## Author Contributions

JW wrote the code, performed the analyses, and wrote the manuscript. AS and PR provided the guidance on methodology and co-wrote the manuscript. MJ supplied the dataset, provided the guidance on methodology, and co-wrote the manuscript.

## Conflict of Interest

The authors declare that the research was conducted in the absence of any commercial or financial relationships that could be construed as a potential conflict of interest.

## Footnotes

^1^The common parlance in psychology and cognitive neuroscience would be “trials,” but the machine learning literature usually says “samples” or “examples.” Given the confusion of using “samples” (since these can also refer to individual data points of EEG), we will use “trials” to refer to these trial-like chunks of neuroscience recording data, in which the intent is to classify each trial/example into one of several categories. One caveat is that our approach relies on combining two “trials” of EEG data to form each “example” for classification, so while “trials” and “examples” would be synonymous for most traditional classification schemes, they are semantically distinct in the context of our PTC approach. Thus, in the present paper, we use “trials” to denote a short chunk of EEG data to be classified, and “examples” to denote the units fed into a classifier, which are either individual “trials” in traditional approaches or pairs of “trials” in PTC.

^2^The method originally used for this dataset by Johnson et al. (2015) was SMLR, but in that paper, the authors were analyzing a different portion of the cognitive task and used a different cross-validation scheme that would not be readily comparable to our PTC approach or other baseline analyses in this paper, so those previous findings are not discussed here.

^3^In some of our preliminary explorations of the PTC concept in another dataset, we tried combining the “trials” end-to-end with various network models, and also tried training SVMs, SMLR, and multilayer perceptrons to perform the same/different classification on similarly concatenated pairs of trials. None of these methods were able to learn to perform the classification above chance, which suggests that structuring the input to “hint” at the paired nature of the problem is necessary for an algorithm to reliably learn the same/different comparison operation. In turn, this means that DNNs, particularly convolutional architectures, may be uniquely well-suited to the task, as these architectures make it much easier to take the structure of the input into account.
